# Spontaneous bleeding of hepatocellular carcinoma during pregnancy

**DOI:** 10.1007/s00404-020-05922-7

**Published:** 2020-12-28

**Authors:** Marco Scioscia, Marco Noventa, Anna Vitulo, Francesca Basile

**Affiliations:** 1 Department of Obstetrics and Gynecology, Policlinico Hospital, Abano Terme, Padua, Italy; 2 Department of Women’s and Children’s Health, Clinic of Gynecology and Obstetrics, University of Padua, Via Giustiniani 3, 3100 Padua, Italy

**Keywords:** Pregnancy, Liver, Carcinoma, Bleeding

Place Fig. 1.

**Fig. 1:**
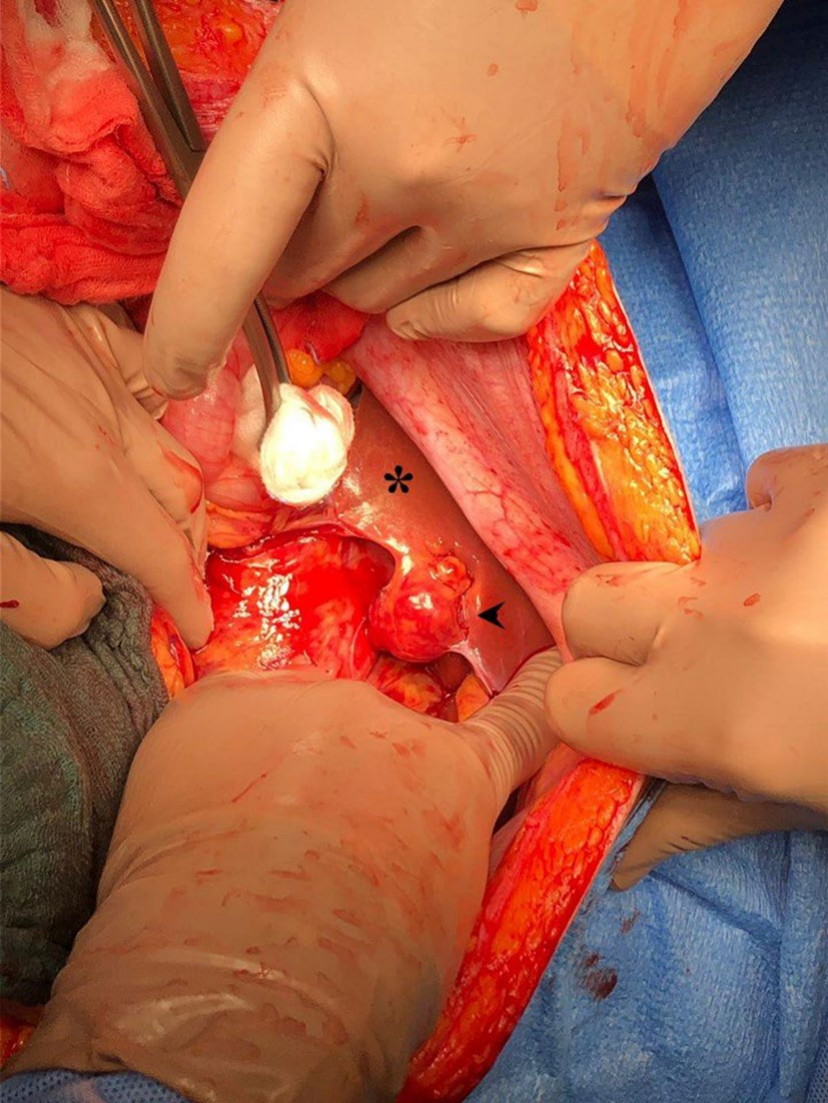
A 31-year-old primigravida at 40 weeks of gestation sought care for onset of severe abdominal pain. Ultrasound revealed a moderate-to-marked amount of hypoechoic free fluid in abdomen. Emergency caesarean section was performed with no evidence of uterine rupture. Active hemorrhage came from a 2.5 cm-sized neoplasm in the right lobe of liver. Nodulectomy was performed and histology confirmed hepatocellular carcinoma. *Normal liver. Arrow—Bleeding neoplasm

